# Supplementary material for: Tunable self-cleaving ribozymes for modulating gene expression in eukaryotic systems
Source: PLoS One. 2020 Apr 30;15(4):e0232046. doi: 10.1371/journal.pone.0232046 (PMC7192461; doi:10.1371/journal.pone.0232046)
Supplement: S1 Fig — (A) Self-cleaving ribozyme that lacks a competing sequence in a cleavable conformation. (B) Self-cleaving ribozyme that contains a competing sequence in a cleavable conformation. (C) Self- cleaving ribozyme that contains a competing sequence in a non-cleavable conformation. The red text indicates the insulating sequence, green text indicates the competing sequence, and black text indicates the ribozyme. (PDF) [file pone.0232046.s001.pdf]

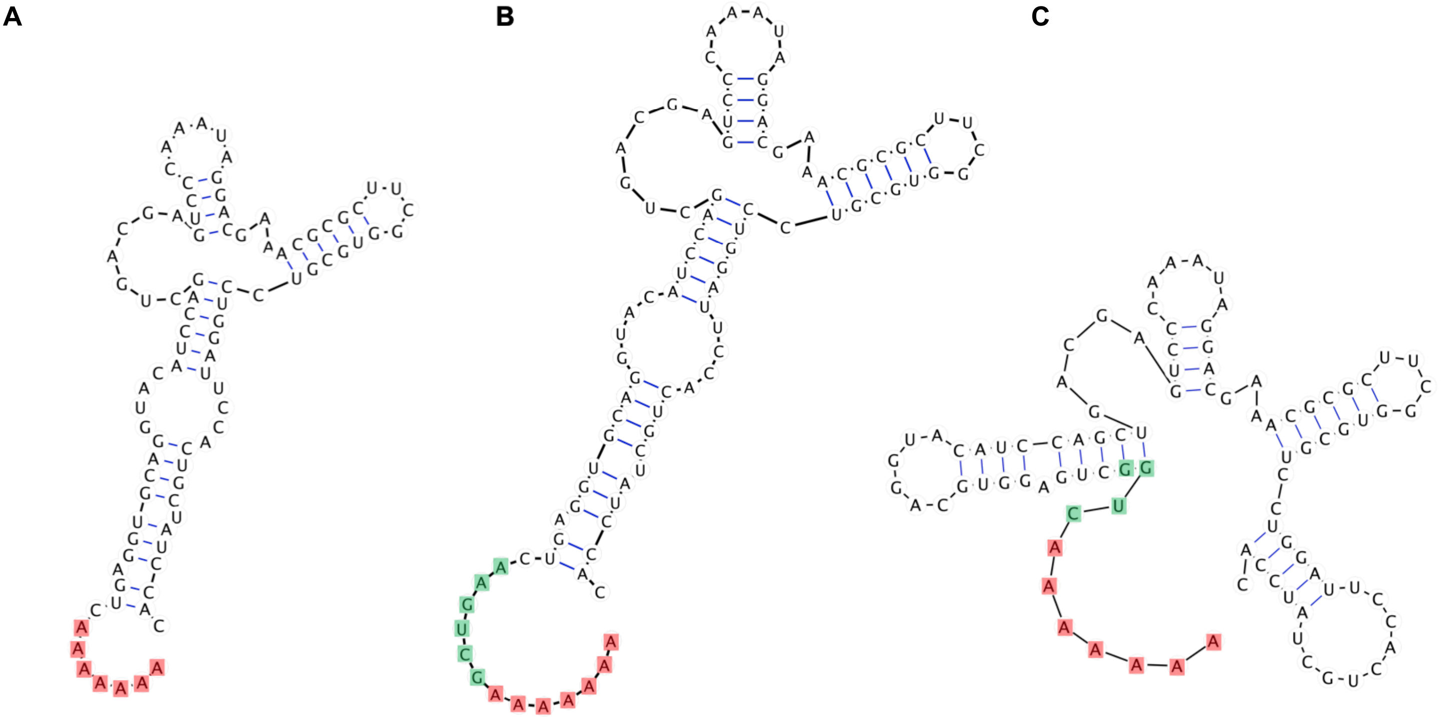

Cleaved ribozyme lacking competing sequence

Cleaved ribozyme with competing sequence

Non-cleaved ribozyme with competing sequence
