# Supplementary figures and images for: Tunable self-cleaving ribozymes for modulating gene expression in eukaryotic systems
Source: PLoS One. 2020 Apr 30;15(4):e0232046. doi: 10.1371/journal.pone.0232046 (PMC7192461; doi:10.1371/journal.pone.0232046)

**A**

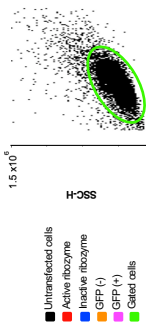

**B**

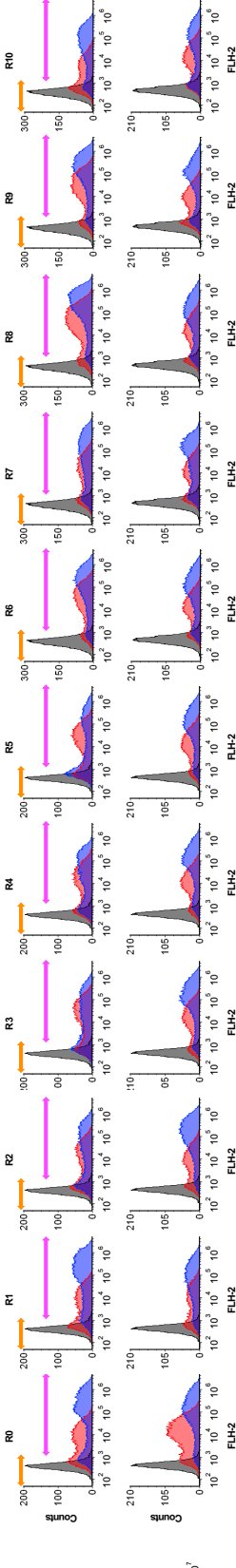

Supplement: S2 Fig — (A) Representative forward and side scatter plot of HEK293T cells transiently transfected with ribozyme constructs. The cell population was gated in green. (B) Histograms of transiently transfected HEK293T cells. Plotted are the number of cells at corresponding fluorescent values of untransfected cells (black), cells containing an active ribozyme/competing sequence (red), and cells containing an inactive ribozyme/competing sequence (blue) in the 5′ untranslated region (top row) or 3′ untranslated region (bottom row) of gfp. (PDF) [file pone.0232046.s002.pdf]

**A**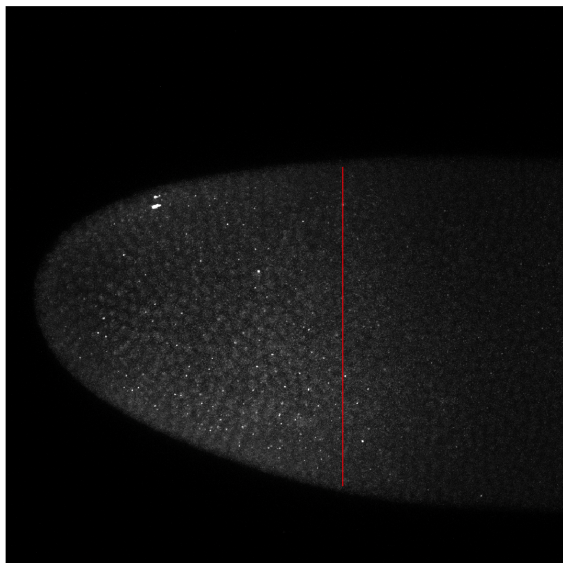**B**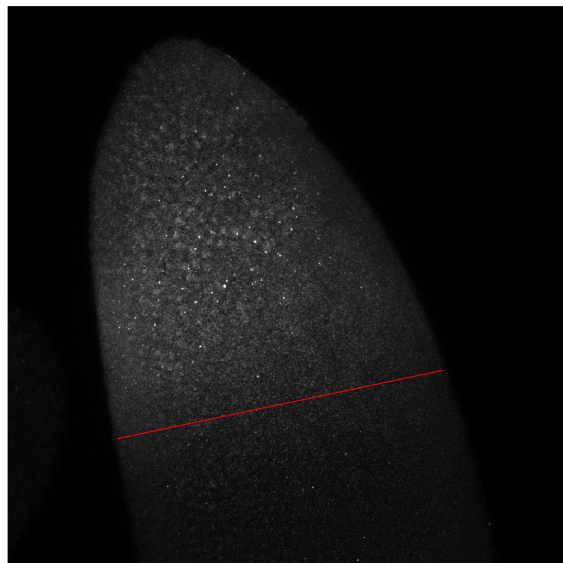**C**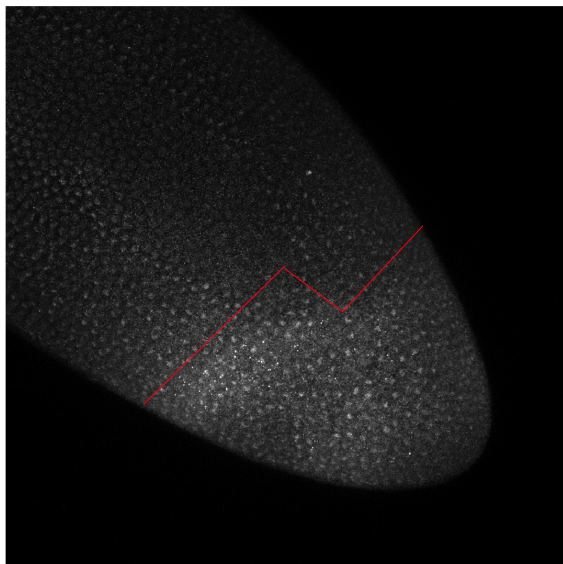**D**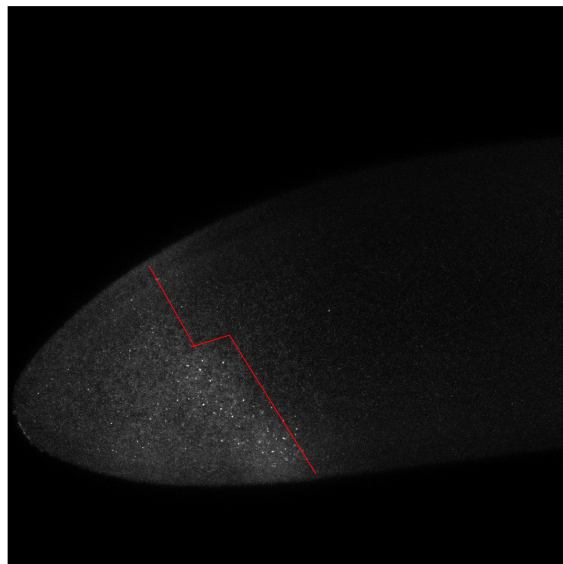

Supplement: S3 Fig — Representative embryos labeled with lacZ gradient width associated with (A/B) symmetric and (C/D) asymmetric lacZ gradients. Red line indicates end of lacZ gradient. Multiple red lines indicate the width of the lacZ gradient at a particular anterior-posterior axis length. (PDF) [file pone.0232046.s003.pdf]

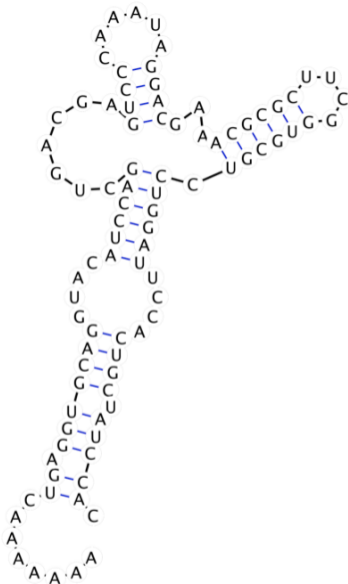

Active Ribozyme

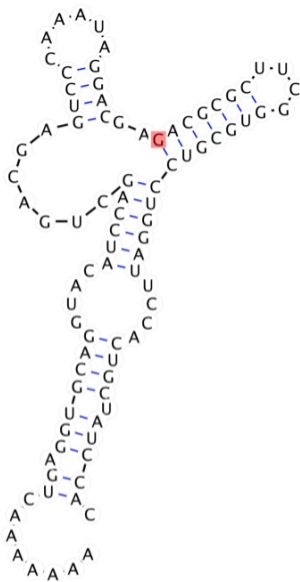

Inactive Ribozyme

Supplement: S4 Fig — The point mutation resulting in loss of self-cleavage is highlighted in red. (PDF) [file pone.0232046.s004.pdf]

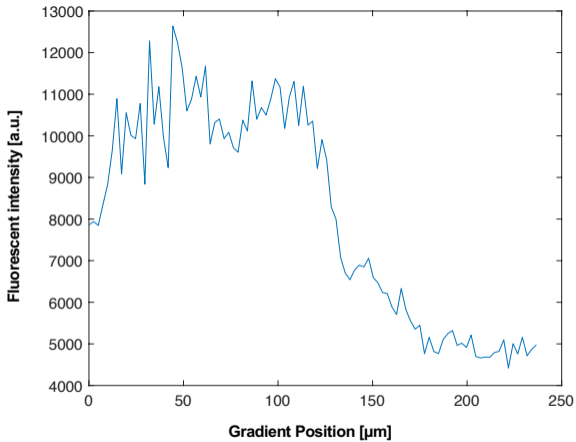

Supplement: S5 Fig — A domain width of zero indicates the anterior pole and increasing values indicate a position closer to the posterior. (PDF) [file pone.0232046.s005.pdf]
